# Advantages of Software Containerization in Public Health Infectious Disease Genomic Surveillance

**DOI:** 10.3201/eid3113.241363

**Published:** 2025-05

**Authors:** Kelsey R. Florek, Erin L. Young, Kutluhan Incekara, Kevin G. Libuit, Curtis J. Kapsak

**Affiliations:** Wisconsin State Laboratory of Hygiene, Madison, Wisconsin, USA (K.R. Florek); State of Utah Department of Health and Human Services, Utah Public Health Laboratory, Taylorsville, Utah, USA (E.L. Young); Connecticut Department of Public Health, Katherine A. Kelley State Public Health Laboratory, Rocky Hill, Connecticut, USA (K. Incekara); Theiagen Genomics, Highlands Ranch, Colorado, USA (K.G. Libuit, C.J. Kapsak)

**Keywords:** public health, SARS-CoV-2, high-throughput nucleotide sequencing, communicable diseases, computational biology

## Abstract

Bioinformatic software containerization, the process of packaging software that encapsulates an application together with all necessary dependencies to simplify installation and use, has improved the deployment and management of next-generation sequencing workflows in both clinical and public health laboratories. Containers have increased next-generation sequencing workflow reproducibility and broadened their usage across different laboratories. We highlight the value of the State Public Health Bioinformatics community’s containerized software repository during the COVID-19 pandemic.

Since 2013, an increasing number of clinical and public health laboratories have adopted next-generation sequencing (NGS)–based assays (*1*). The genomic data generated from NGS assays often require a complex analysis workflow built from a variety of bioinformatic software. Because of the wide range of software used in workflows, challenges can arise when installing software and dependencies, increasing the time and cost of deploying NGS-based tests. Some software distribution tools, such as Conda (https://anaconda.org/anaconda/conda), provide a means to manage the software environment but do not provide an isolated and identical environment and often require additional steps of installing databases and dependencies. The emergence of software container applications has greatly improved NGS workflows by encapsulating software and dependencies into publicly available containers, providing a robust and controlled bioinformatic software solution (*2*,*3*). Ultimately, software containerization simplifies the process of creating and adopting NGS workflows and reduces maintenance issues and downtime, saving time and laboratory resources (*4*).

## Software Containerization

A container is a packaged unit of software that encapsulates an application with all necessary dependencies (*5*). The containers themselves are ephemeral and isolated from both the host environment and other containers. Those qualities ensure that changes occurring within a container are not shared across other containers and that they are not affected by any changes that might occur outside of the container. In that way, using containers increases reliability and reproducibility, even when multiple containers of the same software are running concurrently on the same system.

Software containerization approaches rely on a container engine, such as Docker (https://www.docker.com) or Apptainer (formerly Singularity) (https://apptainer.org), which oversees the tasks associated with creating and using these discrete software environments (*6*). Container images are created using a build file that provides a base image (an initial state for the environment) and stepwise instructions for bundling software code and dependencies. Once the image has been built, it can be used locally or hosted on a public resource where the container can be released and made publicly accessible. Those hosted container images can be downloaded and run on a variety of computing infrastructures, from laptops to high-performance computing (HPC) clusters, greatly simplifying the process of development to deployment (*7*).

**Figure 1:**
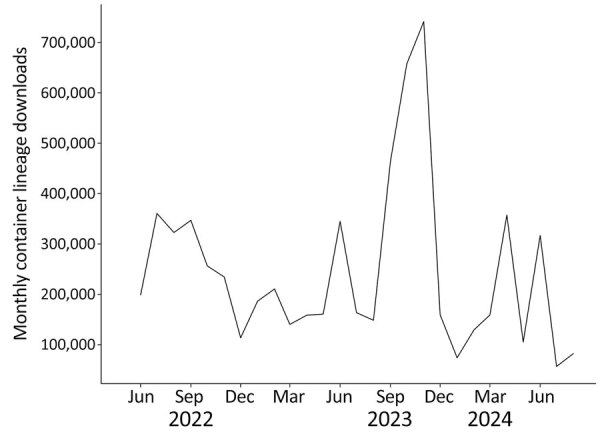
Monthly container image downloads across all State Public Health Bioinformatics containers hosted in Docker Hub (https://hub.docker.com/u/staphb) in study of advantages of software containerization in public health infectious disease genomic surveillance.

Recognizing the value of containers, the State Public Health Bioinformatics (StaPH-B) community developed and actively maintains a repository of containerized software (https://github.com/StaPH-B/docker-builds). The StaPH-B docker-builds repository is a collection of dockerfiles used to build containers of bioinformatics software that are commonly used in public health genomic workflows. Those containers are publicly available and hosted at both Docker Hub (https://hub.docker.com/u/staphb) and Quay.io (https://quay.io/organization/staphb); total monthly downloads from Docker Hub range from 100,000 to >700,000 (Figure 1). The emphasis on quality assurance and quality control in the StaPH-B container repository separates the project from other container repositories. Anyone can submit a new or updated container to the StaPH-B repository following the contributing instructions on the GitHub repository (https://staphb.org/docker-builds/contribute). Each submission includes a test that confirms the functionality of the container image, and all pull requests are reviewed carefully by the repository maintainers to ensure that the software meets the needs and expectations of public health laboratories.

## Containerization in Genomic Workflows

The reliable and reproducible nature of containers is advantageous for public health and clinical laboratories. Deploying bioinformatic analytical workflows and software in a clinical and public health setting is challenging and requires managing computer systems with capacity for scientific computing workloads under regulatory oversight (*8*). Software containerization approaches provide key advantages to genomic workflows.

### Reproducibility 

Containers provide isolated environments that enable strict control of both software versions and software dependencies (*9*). Containers follow a naming convention that is structured as <public-registry-name>/<organization>/<software>:<tag>, which enables quick identification of the software and a specific tag that often indicates version and owner information. In addition, some container engines enable containers to be referenced by a digest, which is a unique and immutable identifier of the container. Referencing containers by the digest ensures the container environment is unchanged and helps support regulatory compliance.

### Isolation

Containers avoid version conflicts and enable multiple versions of software or software dependencies to be used on the same system. In addition, containers provide a separation of data in the container and data on the host system (*6*).

### Replicable and Portable 

Containerized software is easily distributed, reproducible, and readily scalable in Cloud or HPC (*10*). Small databases or reference data can also be incorporated into containers, ensuring the database is maintained and controlled alongside the software.

## Practical Application of Containers during the COVID-19 Pandemic

**Figure 2:**
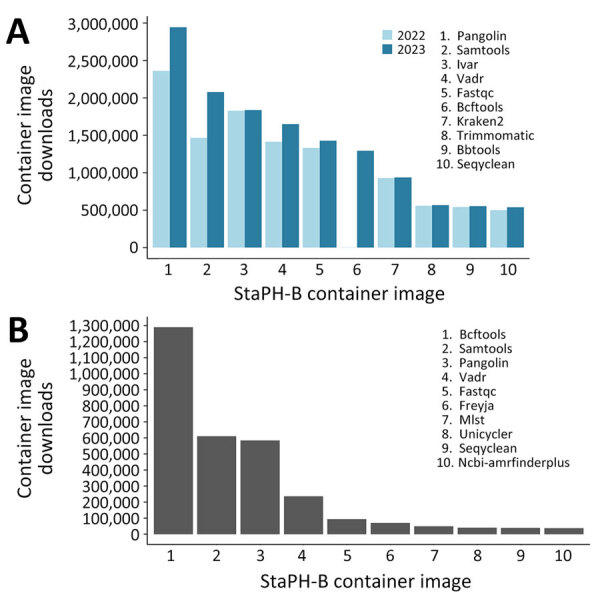
Top container image downloads across State Public Health Bioinformatics containers hosted in Docker Hub (https://hub.docker.com/u/staphb) in study of advantages of software containerization in public health infectious disease genomic surveillance. A) Top 10 overall container image downloads and their downloads in 2022 and 2023. B) Top 10 container images with the largest increase in downloads from 2022 to 2023.

During the beginning of the COVID-19 pandemic, initiatives were created to enhance genomic surveillance (*11*), which led to many clinical and public health laboratories rapidly developing NGS-based surveillance testing to support variant detection. During that time, the StaPH-B docker project played a critical role in supporting the advancement of bioinformatic workflows by providing a reliable resource for SARS-CoV-2 sequence analysis containers. The StaPH-B container of Pangolin, a critical SARS-CoV-2 lineage tool, saw an addition of >500,000 downloads, from 2,360,607 downloads in 2022 to 2,944,235 downloads in 2023 (Figure 2) (*12*). Similarly, the StaPH-B container of iVar, a tool for amplicon-based viral sequencing, has been downloaded >1,836,046 times since it was added in 2020 (Figure 2) (*13*). The scale of downloads from this repository highlights the use of these programs in bioinformatic workflows and the effect of the project during the pandemic.

One of the largest effects on laboratories was a time savings in software installation and management, which grows substantially when scaling workflows to run in an HPC or Cloud environment. Installing Pangolin and its dependencies using Conda takes approximately 3 minutes on a new system, whereas downloading and running the StaPH-B container of Pangolin takes only 1 minute. Similarly, installing iVar takes 4.5 minutes, whereas downloading and running the StaPH-B container of iVar takes only 4 seconds. When using a distributed computing environment such as an HPC or Cloud environment, those time savings become a critical efficiency and cost savings. Those times also assume no installation issues or conflicts with other software and dependencies, a common occurrence with bioinformatics software that can stretch software deployment into days or weeks.

In summary, software containerization has rapidly changed the landscape of bioinformatics over the past 10 years and will continue to be a critical component of public health genomic workflows for the future. The StaPH-B docker project represents a community-based effort providing a valuable resource of standardized bioinformatic tools, supporting reproducibility and regulatory compliance.
